# A pseudovirus-based neutralizing antibody assay for measuring neutralizing antibody against rabies virus

**DOI:** 10.1128/jcm.00877-26

**Published:** 2026-08-21

**Authors:** Xinxin Li, Pei Hu, Yueling Chen, Weizhao Lin, Yingyin Deng, Zixia Qian, Can Xiong, Limei Sun, Jianfeng He, Chunsheng Dong, Jiufeng Sun

**Affiliations:** 1 Guangdong Provincial Institute of Public Health 652472, Guangzhou, China; 2 Guangdong Provincial Center for Disease Control and Prevention, Guangzhou, China; 3 Department of Public Health and Preventive Medicine, School of Medicine, Jinan University 470005, Guangzhou, China; 4 School of Public Health, Guangdong Pharmaceutical University 71237, Guangzhou, China; 5 School of Public Health, Southern Medical University 569910, Guangzhou, China; 6 School of Public Health, Sun Yat-Sen University 71023, Guangzhou, China; 7 Jiangsu Key Laboratory of Infection and Immunity, Institutes of Biology and Medical Sciences, Soochow University 625756, Suzhou, China; Vanderbilt University Medical Center, Nashville, Tennessee, USA

**Keywords:** rabies antibody, rabies vaccine, neutralization assay, pVNA, RFFIT, methodology validation

## Abstract

To establish a laboratory detection assay for a human neutralizing antibody (NAb) against rabies virus and to evaluate its efficiency in human serum samples. A pseudovirus pXN2‑RABV‑G harboring the RABV G gene was applied. The precision, robustness, linearity, stability, inclusivity, and limit of detection of the assay were evaluated. The consistency and correlation of the assay with ELISA and rapid fluorescent focus inhibition test (RFFIT) were analyzed. A pseudovirus-based neutralizing antibody (pVNA) detection system based on the pXN2‑RABV‑G was successfully developed and showed broad robustness (culture media: *χ*^2^ = 4.00, *P* > 0.05, cell passages: *χ*^2^ = 0.25, *P* > 0.05), precision (independent detection: *P* > 0.05), lower/upper limit of linearity (0.09 to 93.73 IU/mL, *R*^2^ > 0.95), and stability (*P* > 0.05). The limit of detection (LOD) was 2.63 IU/mL (95% CI: 1.81–3.84), expressed as the lowest human sera antibody level reliably distinguishable from background signals, and the coefficient of variation was less than 20%. Compared with the commercial ELISA and the gold standard RFFIT assay, the kappa values were 0.74 and 0.82, respectively, and the results were highly consistent. Linear correlation analysis revealed a strong correlation between these variables (*R*^2^ > 0.90). A total of 103 recipients from 2019 to 2023 were included in the serological testing and the following analysis. NAb titers were significantly correlated with vaccine dose (*P* < 0.001) and the most recent vaccination time (*P* < 0.001). The geometric mean titer (GMT) of the 5-dose vaccination group was greater than that of the 1-dose, 2-dose, 3-dose, and 4-dose groups (3.15 vs 3.19 vs 6.18 vs 12.21 vs 13.40 IU/mL). The established pVNA assay for human rabies NAb detection has a detection capability similar to that of the gold standard RFFIT, but it has the advantages of reduced biosafety requirements, laboratory activity, and time, which highlight its safety, high efficiency, and feasibility for screening and monitoring rabies virus antibodies after vaccination.

**IMPORTANCE:**

Rabies is a lethal disease with no effective clinical treatment. Vaccination is the only way to prevent it routinely, which makes post-vaccination antibody testing essential. The current standard test requires a highly equipped laboratory, biosafety measures, and is time-consuming. This study develops a safe, efficient test for detecting rabies neutralizing antibodies using pseudovirus technology. It is comparable to the gold standard test in accuracy but easier to implement in routine labs, which enables broader use in screening and monitoring antibodies post-vaccination. This advantage addresses a key gap in rabies prevention, reducing the uncertainty for individuals with immunodeficiency who are nonresponsive to vaccination and at risk of rabies exposure.

## INTRODUCTION

Rabies is an acute zoonotic infectious disease caused by a neurotropic rabies virus (RABV). Infected people are affected mainly by central nervous system lesions, and the mortality rate is close to 100% (1, 2). RABV belongs to the genus *Lyssavirus* in the family *Rhabdoviridae* of the order *Mononegavirales*. It has a typical bullet shape and is approximately 130–240 nm in length and 65–80 nm in diameter, with a genome size of approximately 12 kb. The RABV genome is sequentially arranged from the 5' end to the 3' end with RNA-dependent RNA polymerase (L), glycoprotein (G), matrix protein (M), phosphoprotein (P), and nucleoprotein (N) (3). Among them, G protein is a transmembrane protein that mediates the binding of the virus to cellular receptors and endocytosis. G protein is the only protein present on the surface and the major antigen to induce neutralizing antibodies (NAb) upon viral infection (4).

Vaccination is the only effective and economical method to prevent rabies (5, 6). The rabies vaccines that have been approved for use in humans are based on inactivated purified RABV grown in tissue culture or embryonated duck eggs/embryonated chicken eggs. Four doses (two doses on Day 0, one dose on Days 7 and 21) or five doses (one dose on Days 0, 3, 7, 14, and 28) comprise the post-exposure (PEP) intramuscular injection vaccination program. At present, in terms of immunogenicity and durable protection after vaccination, existing laboratory detection techniques include the mouse neutralization test (MNT) (7), the “gold standard” rapid fluorescent focus inhibition test (RFFIT) (8), fluorescent antibody virus neutralization test (FAVN) (9, 10), and enzyme-linked immunosorbent assay (ELISA) (11). However, the requirement of RABV culture for these techniques makes them difficult to apply in routine laboratories. Although ELISA is a rapid assay for detecting IgG antibodies against rabies virus and uses a high-throughput, automated method to measure the IgG antibody titer of samples within one day, with a sensitivity and specificity of >90% at titers within its validated detection range, it has serious limitations for practical use. Specifically, the detection range is strictly limited by the assay standard curve, which is typically 5–10 IU/mL for commercial kits, and false-positive or false-negative cases exist for values outside the range. Therefore, there is an urgent need to establish laboratory testing assays targeting NAbs, which can be widely adopted and measure the protective effects of vaccines.

The recombinant vesicular stomatitis virus (rVSV) developed by John Rose and Michael Whitt is widely used as a vaccine vector (12). Especially, rVSVs expressing exogenous viral surface glycoproteins were generated for this kind of application (13–15), indicating the feasibility of rescuing rVSV-based pseudotype viruses. Previously, a rVSV pseudotyped with RABV G protein (VSV-RABV-G) was constructed as a RABV vaccine, and a single dose of VSV-RABV-G vaccination elicited full protection against RABV challenge (16). In the present study, the pXN2‑RABV‑G was further employed for measuring human RABV neutralizing antibodies after vaccination.

The maturity of the novel pseudovirus preparation technology provides important support for the optimization of the pseudovirus-based neutralizing antibody (pVNA) detection system. This method not only has the technical advantages of high biological safety, low cost, and other economic advantages but also can realize standardized operation and large-scale sample testing (Table S1), which makes it possible to gradually replace the traditional detection scheme relying on live virus. The established pVNA assay may provide a potential tool for evaluating RABV vaccination efficacy in routine laboratories.

## MATERIALS AND METHODS

### Cells and virus

BHK-21 cells and 293T cells (GDIPH, Guangzhou, China) were used. The following cell culture media (Gibco, NY, USA) were used: cell culture media (88% DMEM, 10% FBS, 1% HEPES, and 1% PS), cell plating medium (94% DMEM, 4% FBS, 1% HEPES, and 1% PS), and serum and virus diluents (98% DMEM, 1% HEPES, and 1% PS). We previously generated a VSV-RABV-G pseudotype virus for the development of a RABV vaccine (16). The VSV G gene was replaced with the RABV G gene, additionally with GFP insertion, in the VSV-RABV-G viral genome. The virus was propagated in 293T cells.

### Serum samples

Serum samples were obtained from the Guangdong Provincial Vaccination System (GDCDC, Guangzhou, China) between 2019–2023 from post-exposure individuals who initiated PEP with inactivated rabies vaccines.

A total of 120 rabies vaccine recipients were included. All of the vaccine recipients signed an informed consent form (W96-027E-202018). Venous blood samples were collected and transferred to the laboratory of GDIPH at room temperature. Venous blood was centrifuged at 3,000 rpm for 5 min. The supernatant was removed, and the serum was transferred to a 2 mL freezing tube and stored at −80°C.

### RABV_G_ protein recombination, eukaryotic expression plasmid construction, and expression identification

The primers used for the RABV_G_ gene (GenBank: M13215.1) and the expression vector pXN2-GFP-Kana sequences were designed with Primer Premier 6.0 (Premier, CA, USA) without any modification of the enzymatic sites at the 5' and 3' ends. Specifically, forward primer VSV-RABV-GFP F (MluI) and reverse primer VSV-RABV-GFP R (XhoI) were used. Using the RABV plasmid as a template, the target product fragments were obtained via PCR and recovered (Table S2).

**Fig 1:**
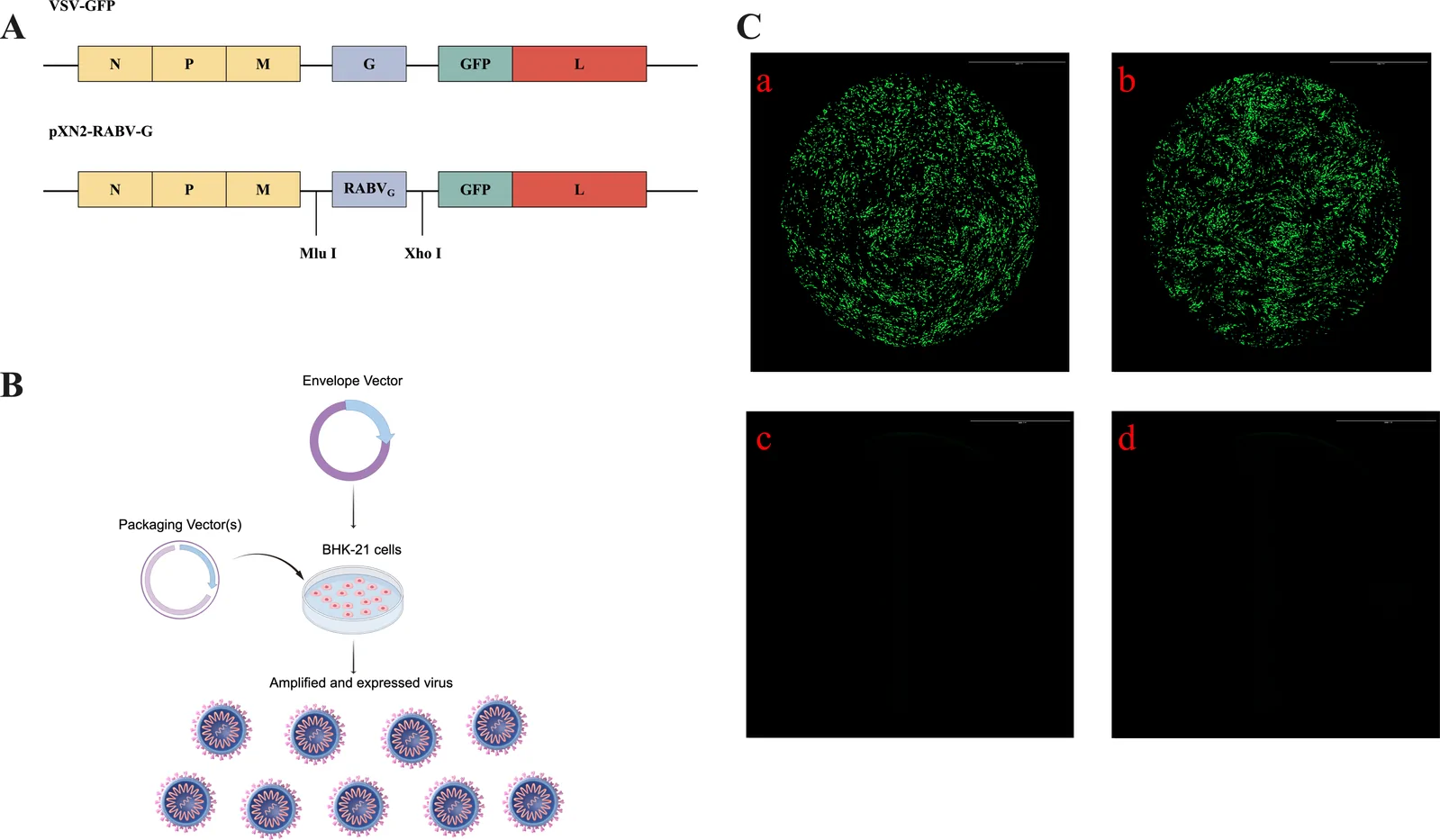
(**A**) Schematic diagram of pXN2-RABV-G construction. (**B**) Schematic diagram of pXN2-RABV-G packaging and transfection. (**C**) Assessment of the serum neutralization ability of pXN2-RABV-G. The cells in the pXN2-RABV-G control (a) and negative control (b) groups showed apparent specific green fluorescence, but none of the positive control (c) or blank control (d) groups were positive. The feasibility of using pXN2-RABV-G for serum neutralization tests was preliminarily demonstrated.

The pXN2-GFP-Kana plasmid was double digested with the restriction endonuclease MluI/XhoI, and 13–15 kb fragments were recovered by separating the fragments using electrophoresis on a 1% agarose gel (RCPSS, Shanghai, China). The RABV_G_ gene DNA fragment was inserted into the expression vector via seamless cloning with the ClonExpress II One Step Cloning Kit (Vazyme Biotech, Nanjing, China) to construct the pXN2-GFP-Kana-VSV-RABV vector (Fig. 1A). The constructed vector was transformed into the competent bacterium *E. coli* DH5α Stable2 (Weidi Biotechnology, Shanghai, China) and then inoculated onto LB agar plates supplemented with kanamycin (RCPSS, Shanghai, China) to select monoclonal colonies for expanded culture.

Briefly, 293T cells were seeded at 5.0 × 10^6^ cells/mL in a 10-cm Petri dish (Corning, NY, USA), cultured at 37°C until the density reached 80%, and then infected with vaccinia-T7 expressing T7 RNA (TaKaRa, Beijing, China) transcriptase for 2 h. Two hours later, four plasmid systems, pXN2-GFP-Kana-VSV-RABV, pP, pL, and pN, were used to cotransfect cells at a ratio of 10:5:4:1 using Lipofectamine 2000 (Thermo Fisher Scientific, MA, USA) and cultured at 37°C with 5% CO_2_. After 48 h, the vaccinia-T7 was removed with a 0.22 μm filter. The recombinant viruses were amplified in 293T cells and purified via gradient centrifugation at 55%, 40%, and 25% sucrose. The cells were resuspended in RIPA lysis buffer (Thermo Fisher Scientific, MA, USA) and lysed on ice for 30 min. After lysis, the cells were centrifuged at 12,000 rpm for 5 min at 4°C. The supernatant was collected, added to 2% FBS medium, and subjected to Western blotting (Bio-Rad, CA, USA) to detect the expression of the RABV_G_ protein, then packaged and stored at −80°C. In this study, the pseudovirus packaged with the RABV_G_ gene was named pXN2-RABV-G (Fig. 1B).

### Concentration and titration of pXN2-RABV-G

The supernatant of the transfected cells was collected for 48 h, the virus mixture was mixed at a ratio of 4:1, the mixture was centrifuged at 1,000 × *g* for 30 min at 2–8°C, and the supernatant was removed to retain the precipitate. The precipitate was resuspended in 1/10–1/100 volume of DMEM to obtain a concentration of 1 × 10^8^ U/mL of pXN2-RABV-G, packaged, and stored at −80°C.

BHK-21 cells were seeded at 1.5 × 10^5^ cells/mL in 96-well plates (100 μL/well) and cultured at 37°C and 5% CO_2_. Seven centrifuge tubes (Corning, NY, USA) were used, and 1,350 µL of pXN2-RABV-G dilution was added to each tube, with six wells per dilution, totaling seven dilutions. Then, 150 µL of pXN2-RABV-G was added to the first 10^−1^ dilution, 150 µL was absorbed from the first dilution to the second dilution gradient, and the mixture was diluted 1:10^−2^ to 1:10^−7^. After the BHK-21 cell density reached 50%–60%, 50 µL of pXN2-RABV-G dilution was added to each well at the corresponding dilution on the plate and cultured at 37°C for 24–48 h. A fluorescence microscope (Huanghe Instrument Technology, Guangzhou, China) (Cat. no. Cytation 5 CYT5PW-SN) was used to observe and record the number of positive wells for fluorescent cells at each dilution. The 50% tissue culture infective dose (TCID_50_) was calculated using the Reed–Muench method.

### Screening and optimization of the pXN2-RABV-G neutralization assay conditions

BHK-21 cells were seeded at 1.5 × 10^5^ cells/mL in 96-well plates (100 μL/well) and cultured at 37°C and 5% CO_2_ until the density reached 50%–60%. Then, they were infected with the pXN2-RABV-G mixture (50 μL/well). Then, 12, 24, 36, 48, 60, and 72 h after pXN2-RABV-G virus infection, the fluorescence spot was detected, and a curve of the fluorescence spot with respect to time was drawn to determine the fluorescence signal platform and the best experimental detection time.

Furthermore, BHK-21 cells were seeded and cultured under the same conditions. pXN2-RABV-G was diluted to 50, 100, 200, 400, 800, and 1,600 TCID_50_/50 μL, after which BHK-21 cells were infected (50 μL/well). According to the selected optimal detection time, the fluorescent spot was detected, and the curve of the fluorescence spot based on the pXN2-RABV-G dilution gradient was drawn to determine the optimal pXN2-RABV-G dosage in the linear range.

### Validation of pVNA

#### Precision

(i) Repeatability: Ten positive serum samples for rabies virus neutralizing antibodies were tested 10 times via pVNA, and the coefficient of variation (CV%, CV% = $\frac{S}{\bar{x}}$ × 100%) was calculated. (ii) Intermediate precision: The same samples were tested six times in six days by an operator, and the differences between the samples tested by the operator were analyzed. The same samples were tested independently six times over six days, and the differences between different operators were analyzed.

#### Robustness

Eight serum samples with different neutralizing antibody titers (NAb titers) (IU/mL) were tested, and the effects of culture media (medium within 1, 6, and 12 months after production) and cell passages (3, 5, and 7 passages) on the NAb titers of the samples were analyzed. The assay was repeated three times, and the $\bar{x}$ ± s, CV%, and GMT were calculated.

#### ULOQ and LLOQ

The upper limit of quantification (ULOQ) and lower limit of quantification (LLOQ) of the pVNA were determined using the Third WHO International Standard for Anti-Rabies Immunoglobulin (NIBSC 19/244), a reference reagent with a certified potency of 164 IU per ampoule validated by the gold-standard RFFIT assay. The stock standard (164 IU/mL) was two-fold serially diluted in matrix-matched buffer into nine concentrations (0.64 to 164 IU/mL). Each concentration was tested across four replicates following standard pVNA procedures, and fluorescence response signals were collected. Data outliers beyond mean ± 2 standard deviations (SD) were excluded. Dose-response data were fitted with the four-parameter logistic (4PL) model. The response signal at which the pVNA median effective dose (ED_50_) was calculated is as follows (17). ED_50_ is defined as the immunoglobulin dilution producing fluorescence signals equivalent to 50% virus inhibition:


$$\begin{array}{rl} & pVNA\;ED_{50}=Bottom+\frac{Top-\text{ Bottom }}{1+10^{\left(\log{EC}_{50}-X\right)\times \text{ Hillslope }}} \end{array}$$

where *X* = log(concentration).

The valid pVNA ED_50_ criteria were as follows: intra-assay CV ≤10%, and *R*^2^ should be ≥0.95. After curve evaluation for plateau saturation and baseline stability, the maximum valid ED_50_ value was assigned as ULOQ and the minimum stable baseline ED_50_ as LLOQ; an extra safety margin was reserved adjacent to the linear boundary to eliminate quantification bias from extreme signal saturation.

#### Linearity

Linear regression was conducted across the 4PL-validated quantifiable range to assess assay linearity. The maximum concentration for linear analysis was set to the WHO standard concentration corresponding to the ULOQ derived from the 4PL model. Log_10_-transformed WHO standard concentrations (independent variable) and log_10_ pVNA ED_50_ values (dependent variable) were tested per standard pVNA protocols. Acceptance criteria cited from reference (18): *R*^2^ ≥ 0.95, slope 95% CI between 0.70–1.43, intra-assay %GCV ≤ 10%, and acceptable relative bias for all dilutions. The upper/lower limit of linearity was defined as the highest/lowest standard concentration meeting all linear criteria with no saturation or residual bias.

#### Stability

To assess the freeze/thaw stability of the serum samples for the assay, 21 positive serum samples for rabies virus neutralizing antibodies were aliquoted, stored at −80 ± 10°C, and then exposed to a series of 1–5 additional freeze/thaw events. The MN50 values of fresh samples were used as a baseline for comparison in three assay runs. The target criterion was that the percent recovery should be within −50% to 100% for at least 80% of samples (18).


$$Recovery=\frac{\text{Fresh samples MN50-Freeze/T}\text{haw samples MN50}}{\text{Fresh samples MN50}}\times 100\%$$

#### Inclusivity

Ten positive serum samples for rabies virus neutralizing antibodies from Panyu and Heyuan and eleven positive serum samples from Panyu from 2020 to 2023 were tested in three runs. Then, the CV% was calculated.

#### Limit of detection

Four positive serum samples for rabies virus neutralizing antibodies from different sources were diluted twofold from 1:64 to 1:8,192, and each diluent was tested in twelve runs. The virus inhibition rate of 50% per dilution was used as the positive detection limit, and the antibody level GMT with a 95% positive detection rate was used as the limit of detection.

### pXN2-RABV-G serum neutralizing antibody (pVNA) assay

BHK-21 cells were seeded at 1.5 × 10^5^ cells/mL with 4% FBS cell planting medium in 96-well plates and cultured at 37°C and 5% CO_2_. Serum samples were serially diluted 1:8 to 1:4,096 with serum diluent, for a total of nine dilution gradients, and each gradient was set up in two wells. A total of 800 TCID_50_/50 μL of pseudovirus mixture was added to each well. The sample mixture was mixed with the pseudovirus mixture in equal volumes, and a cell control and a pseudovirus control (VC) were used. The pseudovirus serum mixture was inoculated into preplated BHK-21 cells (100 μL/well). After 48 h, the number of fluorescent spots was automatically counted using a fluorescence microscope imaging system, and the viral infection inhibition rate was calculated. Neutralizing antibody (NAb) titer was defined as ED_50_ that inhibited ≥50% of fluorescent spots compared with the viral control after conversion to the WHO standard.


$$Viral\;infection\;inhibition\%=1-\frac{\text{Serum spots-CC spots}}{\text{VC spots-CC spots}}\times 100$$

### Rabies virus antibody (human) ELISA

A rabies virus antibody (human) ELISA (DRG Instruments GmbH, Marburg, GER) (Cat. no. EIA-5900) was used to detect RABV anti-G protein IgG in the serum samples. The negative and positive controls were reconstituted directly with 0.5 mL/1 mL deionized water. The experimental procedure was conducted strictly according to the kit’s manual instructions. The absorbance values at 450 nm were read at 620 nm as a reference on the ELISA reader. The optical density (OD) value was entered into SigmaPlot software to construct a curve, and the sample ELISA titer was calculated according to the OD on the Y-axis and the titer on the X-axis of the critical line.

### RFFIT

The rapid fluorescent focus inhibition test (RFFIT) is the standard method recommended by WHO to detect rabies virus neutralizing antibodies (RVNA). The principle of the test is to use the standard serum as the internal reference and the Third WHO International Standard for Anti-Rabies Immunoglobulin (19/244) as the external reference. The quantitative rabies virus was neutralized with serial dilutions of the tested serum, and the unneutralized virus was inoculated into susceptible cells. The rabies virus was stained with a fluorescent antibody against rabies virus nucleoprotein, and the residual virus was detected by fluorescent foci. Finally, the neutralizing antibody ED_50_ of the serum samples was calculated according to the neutralization test calculation formula, taking the standard serum antibody ED_50_ as the reference. WHO considers that a virus neutralizing antibody level of ≥0.5 IU/mL after vaccination confers effective protection.

### Calibration and bridging experiment using WHO international standard

To convert pVNA ED_50_ from dilution fold to international units (IU/mL), a calibration and bridging experiment was performed using the Third WHO International Standard for Anti-Rabies Immunoglobulin (NIBSC code: 19/244). A working standard solution was prepared at a calibrated concentration of 11 IU/mL and subjected to 3-fold serial dilutions with six repetitions. A conversion fold between pVNA dilution fold and IU/mL was established based on the calibrated standard. This conversion enables direct and clinically relevant comparison of pVNA results with the WHO-recommended protective threshold of 0.5 IU/mL.

### Accuracy study

The pVNA established in this study was compared with rabies virus antibody (human) ELISA and the gold standard rapid fluorescent focus inhibition test (RFFIT) in 120 post-exposure recipients vaccinated against rabies. The positive/negative percent agreement, sensitivity, specificity, Youden index, and kappa value were calculated to analyze the consistency of the detection assay. 120 unvaccinated individuals were tested for the pVNA and RFFIT to determine the specificity of the assay. A simple linear regression was performed with the sample ED_50_ of pVNA (IU/mL) as the independent variable and the sample ED_50_ of ELISA/RFFIT (IU/mL) as the dependent variable to analyze the correlation between assays. Receiver operating characteristic (ROC) curve analysis was performed using pVNA results (positive/negative) as the test variable and RFFIT results (positive/negative) as the gold-standard classification.

### Statistical analysis

All the data were analyzed via RStudio 4.2.2 (Posit Software, Boston, MA, USA) and GraphPad Prism 10.0.1 (GraphPad Software, San Diego, CA, USA). The generalized additive model (GAM) was used for the antibody attenuation model. Student’s, Wilcoxon, Friedman, Mann–Whitney, and Kruskal–Wallis tests were used for the significance tests. The test level was α = 0.05, and *P* < 0.05 was considered statistically significant.

## RESULTS

### Optimization of pVNA assay conditions

**Fig 2:**
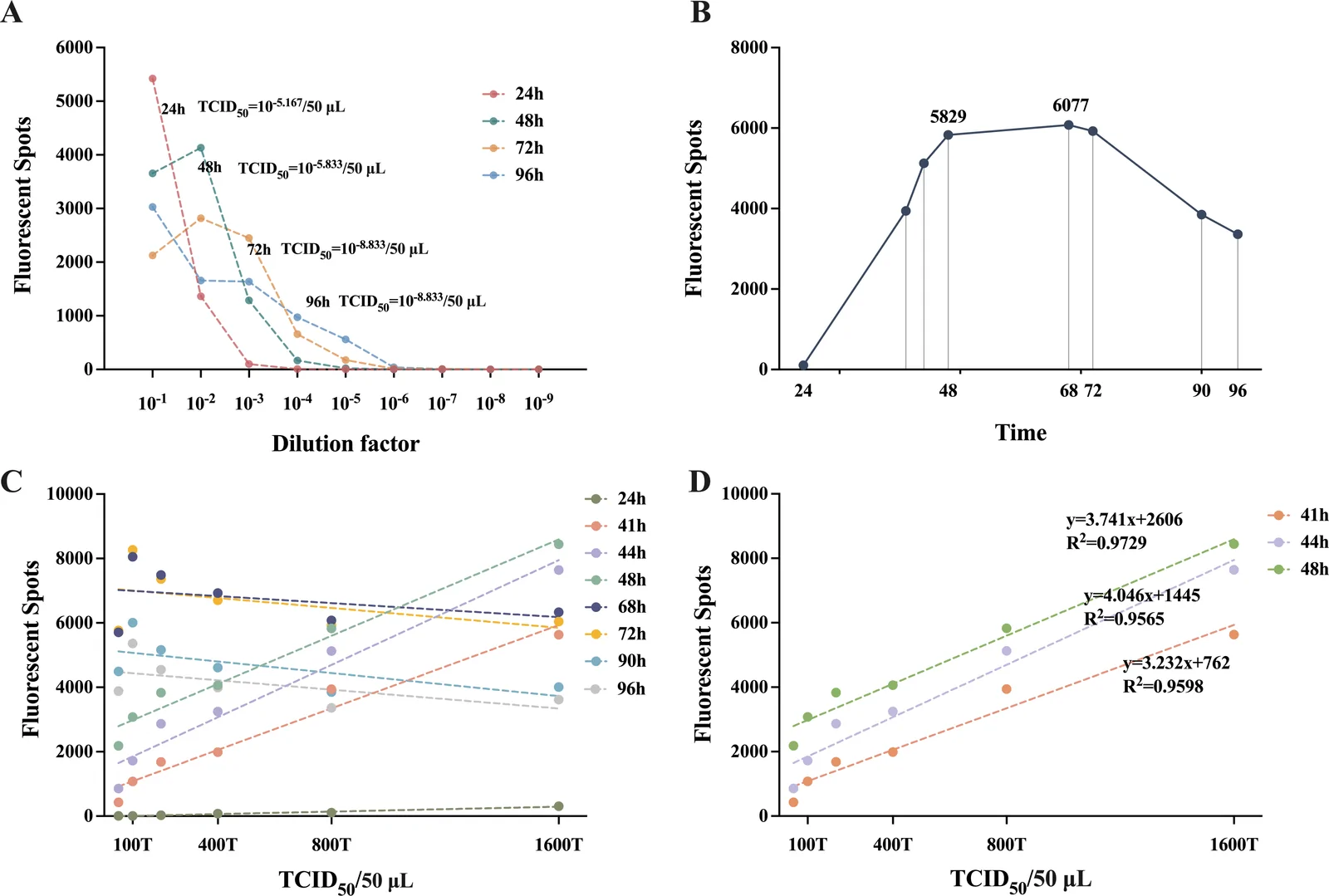
(**A**) Effect of incubation time on the pXN2-RABV-G titer. (**B**) Effect of incubation time on the number of fluorescent spots. (**C**) Effect of the pXN2-RABV-G dose on the number of fluorescent spots. (**D**) The pXN2-RABV-G dose was linearly correlated with the number of fluorescent spots.

In the pXN2-RABV-G viral genome, the VSV G protein was replaced with RABV G for cell surface binding and entry (Fig. S1). Additionally, a GFP reporter gene was inserted following RABV G (Fig. 1A). BHK-21 cells were infected with pXN2-RABV-G at a multiplicity of infection of 10, and a cytopathic effect was observed at 2 days postinfection, with specific green fluorescence detected in infected cells under the fluorescence field (Fig. 1C). For the pXN2-RABV-G infection within 96 h, the number of fluorescent spots increased up to 48 h. After 48 h, the number reached a plateau and decreased (Fig. 2A and B; Fig. S2).

48 h was chosen as the detection time for pVNA. Compared with the traditional neutralizing antibody detection assay, the experimental cycle was greatly shortened, and the efficiency was improved.

The curve of the fluorescent spots with pXN2-RABV-G dilution is shown in (Fig. 2C). The results revealed a good linear relationship between the pXN2-RABV-G TCID_50_/50 μL ratio and the number of fluorescent spots from 41 to 48 h of incubation (Fig. 2D). In this study, to ensure the validity of the experimental results, 800 TCID_50_/50 μL was selected as the optimal dosage of pVNA (Table S3).

### Validation of pVNA

The CV% of the repeatability test of 10 positive serum samples was less than 20%, indicating good repeatability (Table S4). No significant differences in NAb GMT in the same serum sample were detected by the operator at different times (operator A: *P* > 0.05; operator B: *P* > 0.05) (Table S5). No significant differences in the NAb GMT of the same samples were detected independently (*P* > 0.05) (Table S6). The established assay showed good intermediate precision in detecting NAb GMT.

No significant differences in the NAb GMT of eight serum samples were observed when the medium was assessed 1, 6, and 12 months after production (Table S7), indicating that the medium had no effect on the test results (*χ*^2^ = 4.00, *P* > 0.05). In addition, there was no significant difference in the NAb GMT among the eight serum samples when three, five, or seven cell passages were used (*χ*^2^ = 0.25, *P* > 0.05).

**Fig 3:**
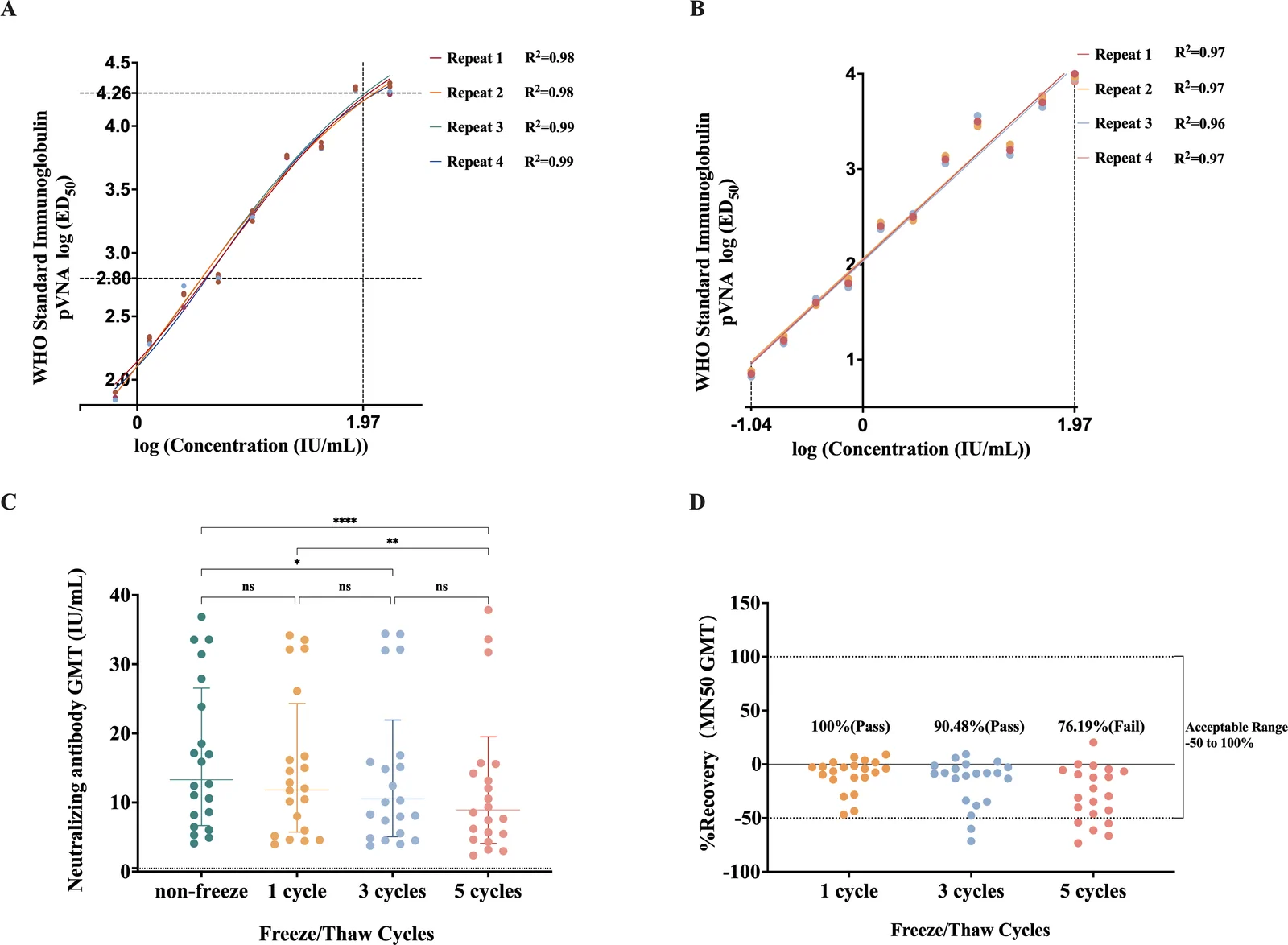
(**A**) 4PL sigmoidal fitting curves of serially diluted WHO standard immunoglobulin (*R*^2^ ≥ 0.98). Valid ED_50_ quantitation range: log ED_50_ 2.80-4.26. (**B**) Linear regression for linearity assessment (*R*^2^ ≥ 0.96). The linear range spanned 0.09–93.73 IU/mL (log concentration −1.04 to 1.97). (**C**) Effects of sample freezing/thawing on the serum sample titer and freeze/thaw stability of the serum samples for the microneutralization assay (*N* = 21) (ns, no significance). (**D**) The acceptable deviation range was −50% to 100% of the reference condition values (shown by dashed lines).

Nine concentrations of standard immunoglobulin were positively correlated with the pVNA log ED_50_, yielding sigmoidal curves suitable for four-parameter logistic (4PL) fitting (Fig. 3A). Fitting of the 4PL model produced coefficients of determination *R*^2^ ≥ 0.98, enabling robust calculation of the ULOQ and LLOQ (Table S8). The ULOQ was set at log ED_50_ = 4.26 for the optimal platform state and four replicates, and the intra-assay CV% was within the acceptable range, close to the 90% platform state. The higher value caused saturation and poor precision. Baseline log ED_50_ = 2.80 was defined as LLOQ, and the within-run CV% was acceptable. To characterize assay linearity, log-transformed WHO standard immunoglobulin concentrations and matched log pVNA ED_50_ values were subjected to linear regression (Fig. 3B). The linear model met all predefined validation criteria: *R*^2^ ≥ 0.96, slope 95% CI within 0.70–1.43, and intra-assay %GCV ≤10%. The log concentration linear range from −1.04 to 1.97, equivalent to a standard concentration from 0.09 to 93.73 IU/mL, was identified as the range.

A significant difference in NAb GMT was noted between nonfrozen samples and freeze-thaw samples (*P* < 0.05) (Fig. 3C; Table S9). Taking the MN_50_ of the nonfrozen samples as the baseline, more than 80% of the MN_50_ values of the 1- and 3-cycle samples were in the acceptable range of −50% to 100%, whereas only 76.19% of the MN_50_ values of the 5-cycle samples were in the acceptable range. These results indicate that the number of sample freeze/thaw cycles should not exceed three (Fig. 3D). No significant differences in NAb GMT were noted between different geographical samples (Heyuan vs Panyu, *P* > 0.05) (Table S10), and no significant differences in the NAb GMT were noted among the samples collected in 2020, 2021, 2022, or 2023 (*P* > 0.05). Based on repeated testing of four positive samples, the limit of detection (LOD) was 2.63 IU/mL (95% CI: 1.81–3.84). (Table S11).

### Agreement among the pVNA, ELISA, and RFFIT assays

A total of 120 rabies post-exposure vaccinated recipients (full cohort) from 2019 to 2023 were included. Ultimately, 13 recipients with incomplete vaccination information were excluded. Serological testing was performed on 107 subjects. Four recipients with negative antibodies in the serum test were excluded based on the internally derived pVNA cutoff determined by the calibration and bridging experiment, and 103 recipients with positive antibodies against the rabies vaccine were finally descriptively analyzed (Table 1). There was no significant difference in NAb titer according to sex or age (39.35 ± 20.41 years) (*P* > 0.05). A total of 10 (9.71%), 10 (9.71%), 8 (7.76%), 22 (21.36%), and 53 (51.46%) patients received 1, 2, 3, 4, and 5 doses of a rabies vaccine, respectively. A total of 12 (11.65%), 31 (30.10%), 31 (30.10%), 27 (26.21%), and 2 (1.94%) patients received the rabies vaccine in 2023, 2022, 2021, 2020, and 2019, respectively. The results revealed that NAb titers were significantly correlated with vaccine dose (*P* < 0.001) and the most recent vaccination time (*P* < 0.001). The GMT of the 5-dose vaccination group was greater than that of the other groups.

**TABLE 1:** Demographic and epidemiological information of 103 subjects

Parameter	*N* (%)	GMT (IU/mL)	T/F	*P*
Geographical location				
GuangZhou	37 (35.92%)	3.15	0.95	0.62
LeiZhou	33 (32.04%)	3.19		
HeYuan	33 (32.04%)	6.18		
Sex				
Male	52 (50.48%)	10.76	1,321	0.97
Female	51 (49.52%)	10.46		
Age (years)	39.35 ± 20.41^*a*^			
≤20	14 (13.59%)	10.76	2.66	0.62
21–40	46 (44.66%)	9.78		
41–60	24 (23.30%)	11.11		
61–80	14 (13.59%)	12.87		
≥80	5 (4.86%)	9.15		
Vaccination (doses)				
1	10 (9.71%)	3.15	19.03	<0.001^**b^
2	10 (9.71%)	3.19		
3	8 (7.76%)	6.18		
4	22 (21.36%)	12.21		
5	53 (51.46%)	13.40		
Time of last vaccination (year)				
2023	12 (11.65%)	18.21	24.20	<0.0001^****^
2022	31 (30.10%)	14.38		
2021	31 (30.10%)	8.42		
2020	27 (26.21%)	5.84		
2019	2 (1.94%)	5.07		

^*a*^ Age was normally distributed and is presented as the means ± standard errors (SEM).

^*b*^ ** indicates *P* < 0.01 and **** indicates *P* < 0.0001.

**Fig 4:**
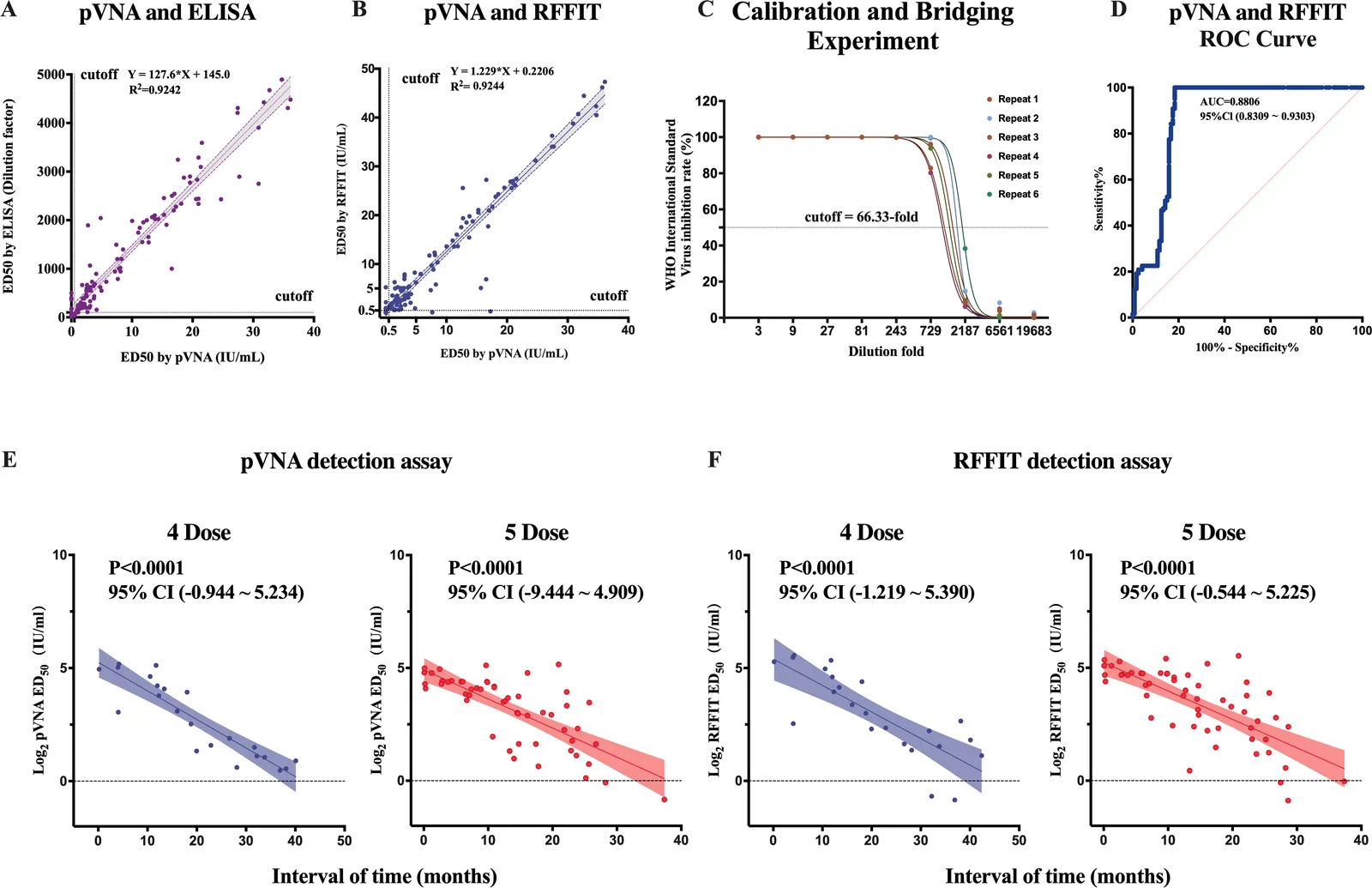
(**A, B**) pVNA NAb ED_50_ correlated linearly with ELISA/RFFIT ED_50_. (**C**) Calibration assay with WHO International Rabies Antibody Standard, with the derived cutoff value marked. (**D**) ROC curve analysis of pVNA against the RFFIT reference. (**E, F**) Trends in the decay over time of NAb ED_50_ in post-exposure recipients vaccinated against rabies, depicting four and five doses vaccination, using the pVNA and RFFIT detection assays, respectively.

For method comparison, all 120 samples were tested by the pVNA assay, ELISA, and the gold-standard RFFIT, meeting quality criteria for all assays. Sufficient statistical power supported 2 × 2 contingency table analysis and reliable agreement assessment. Compared with ELISA, pVNA showed a significant difference (*P* < 0.001), high accuracy (Youden index = 0.81, 95% CI: 0.49–0.94), and high consistency (kappa = 0.74, 95% CI: 0.65–0.83), with a strong linear correlation (*R*^2^ > 0.92) (Table 2; Fig. 4A).

**TABLE 2:** Consistency between the pVNA and IgG ELISA results

pVNA	IgG ELISA		Total
	Positive	Negative	
Positive	101	2	103
Negative	5	12	17
Total	106	14	120
Positive percent agreement (%)	95.28% (95% CI: 89.43%–97.97%)		
Negative percent agreement (%)	85.71% (95% CI: 60.05%–95.99%)		
Youden index	0.81 (95% CI: 0.49–0.94)		
Agreement rate (%)	94.17% (95% CI: 88.45%–97.15%)		
*P* value of *χ*² test	<0.001		
Kappa score	0.74 (95% CI: 0.648–0.834)		
*P* value of kappa consistency test	<0.001		

Compared with RFFIT, pVNA showed high sensitivity (96.23%, 95% CI: 91.19%–98.72%), specificity (92.86%, 95% CI: 66.51%–99.82%), and excellent consistency (kappa = 0.82, 95% CI: 0.71–0.93), also showing a strong linear correlation (*R*²>0.92) (Table 3; Fig. 4B). In addition, pVNA specificity was evaluated in 120 unvaccinated healthy individuals that all RFFIT-negative, with all results negative (Table 4). Specificity was 100% (95% CI: 97.02%–100%), false-positive rate 0%. No positive samples precluded sensitivity and Youden index calculation. pVNA accurately identified all negative sera, showing excellent specificity.

**TABLE 3:** Consistency between the pVNA and RFFIT (gold standard) results

pVNA	RFFIT (gold standard)		Total
	Positive	Negative	
Positive	102	1	103
Negative	4	13	17
Total	106	14	120
Sensitivity (%)	96.23% (95% CI: 91.19%–98.72%)		
Specificity (%)	92.86% (95% CI: 66.51%–99.82%)		
Youden index	0.90 (95% CI: 0.75–0.95)		
Agreement rate (%)	95.83% (95% CI: 90.74%–98.59%)		
*P* value of *χ*² test	<0.001		
Kappa score	0.82 (95% CI: 0.71–0.93)		
*P* value of kappa consistency test	<0.001		

**TABLE 4:** Specificity of the pVNA assay for Rabies virus antibody in all negative serum

pVNA	RFFIT (gold standard)		Total
	Positive	Negative	
Positive	0	120	120
Negative	0	120	120
Total	0	120	120
Sensitivity (%)	NA		
Specificity (%)	100% (95% CI: 97.02%–100%)		

To enable standardized and clinically comparable reporting, the 11 IU/mL standard was serially diluted by pVNA, reaching ED_50_ at 1,450.5-fold. Calibration yielded 1 IU/mL = 132.67-fold dilution, so the WHO 0.5 IU/mL threshold corresponds to a pVNA cutoff of 66.33-fold, allowing IU/mL reporting (Fig. 4C). ROC curve analysis (RFFIT as reference) gave an area under the curve (AUC) of 0.88 (95% CI: 0.83–0.93), confirming the favorable diagnostic performance (Fig. 4D).

### GAM diagnostics and antibody decay kinetics

GAM diagnostics (Fig. S3) validated the robustness and adherence to key assumptions of the antibody decay models across all subgroups. Residual analysis revealed symmetric zero-centered distribution without systematic bias or trends, confirming no critical effect omission. Homoscedasticity was confirmed by constant residual variance, while Q-Q plots verified approximate normality of residuals. Residual-order plots excluded autocorrelation, eliminating time-dependent confounding.

Time-trend analysis of NAb ED_50_ post-vaccination demonstrated congruent decay profiles between the pVNA and gold-standard RFFIT (Fig. 4E and F). In four-dose and five-dose fully vaccinated cohorts, NAb ED_50_ exhibited a significant negative correlation with the interval between last vaccination and sampling (*P* < 0.0001). Linear decay parameters (95% CI): for the 4-dose group, pVNA presented an intercept of 5.25 and slope of −0.13 (95% CI: -0.15–0.10), whereas RFFIT yielded an intercept of 5.4 and slope of −0.12 (95% CI: -0.16-–0.08). For the 5-dose group, pVNA showed an intercept of 4.91 and slope of −0.13 (95% CI: -0.16-–0.10), while RFFIT had an intercept of 5.23 and slope of −0.13 (95% CI: -0.16–0.93).

All GAM models yielded a smoothing parameter of 1, indicating strictly linear decay trajectories. According to the GAM, the NAb ED_50_ (IU/mL) detected by pVNA was 10.31 IU/mL (95% CI: 7.88–13.51), 3.53 IU/mL (95% CI: 2.06–6.05), 1.20 IU/mL (95% CI: 0.54–2.71) at 12, 24, and 36 months after complete vaccination (five doses), respectively, and declined to the cutoff value 3.8 yr later. Concordant kinetic parameters and overlapping CIs between pVNA and RFFIT corroborate that the established pVNA assay reliably recapitulates the long-term decay kinetics of vaccine-induced neutralizing antibodies. The trend of antibody attenuation can be used as a reference to assess the optimal collection time after post-exposure in the local population to ensure adequate antibody development.

## DISCUSSION

Biosafety level (BSL)-3/4 facilities are required for the assessment of highly pathogenic pathogens, and these facilities are not readily available for most of the global R&D community. Thus, developing non-pathogenic pseudotyped viruses is an alternative solution to facilitate basic and serological studies of highly pathogenic pathogens in BSL-1/2 laboratories. HIV-1, VSV, and MLV packaging systems are the main systems used for RABV pseudovirus packaging (19). In this study, we provide an infectious rVSV pseudotyped with RABV G for the assessment of rabies vaccination efficacy.

pXN2‑RABV‑G carries the GFP fluorescent gene for convenient detection (20, 21), which could play a unique role in antiviral drug research and vaccine efficacy evaluation. The assay was initially validated according to the standards issued by the National Medical Products Administration, the Chinese Pharmacopeia Commission, and the World Health Organization (WHO) (22–31). Our results showed good repeatability, intermediate precision, and inclusivity. These results are similar to the findings of Moura et al. (32) and Šuštić et al. (33, 34) and also comparable to the linear range of other antibody assays (35–37).

The time-resolved fluoroimmunoassay (TRFIA) is based on a sandwich-type immunoassay and is similar to the ELISA. The RFFIT assay uses focal inhibition of antibody reactions to detect RABV antibodies. The WHO recommends the use of RFFIT for detection in human samples, and this method serves as the gold standard (38). FAVN is recommended by the World Organization for Animal Health (WOAH) for detecting RABV antibodies in animal serum (39). The correlation coefficient of nucleoprotein values between the novel TRFIA and ELISA was 0.981 (40), and that of titer values between TRFIA and RFFIT was 0.733 (41). The kappa statistic between RFFIT and in-house ELISA in the Thangavelu study (42) was 0.99 (95%: CI 0.95–1.00). Some studies have reported a strong correlation between ELISA and FAVN results in tested animal samples (*r* = 0.92) (43). In fox samples, the overall coincidence rate between the two assays was 65.66%, and 70% of the samples were consistent between the two assays (44). A high concordance (95%) was observed between the ELISA and the FAVN assay, which was similar in sera from red foxes and raccoon dogs (45). Collectively, these studies verify that G-protein-targeted neutralizing antibody assays generally show good intermethod correlation.

This study compared pVNA with the gold standard RFFIT and reported a correlation between them and high agreement. Furthermore, according to the RABV neutralizing antibody determined in the RFFIT, titers of ≥0.5 IU/mL are considered adequate for rabies protection (46). A limitation of this assay is that the titers are not expressed in regular IU/mL, in contrast to that noted for the RFFIT or FAVN assays. This assay can be combined with the currently used international standard of rabies immune globulin for titer conversion to IU/mL (47). Interpreted according to the 0.5 IU/mL protective threshold calibrated by the WHO international standard, the pVNA assay demonstrated 100% specificity with no false-positive detections in unvaccinated healthy seronegative populations. Such excellent analytical performance was attributed to the specific immunological recognition of rabies virus glycoprotein G, which excluded non-specific cross-reactive interference. Among vaccinated individuals, a specificity of 92% was identified, arising from only one discrepant sample with antibody levels clustering closely around the diagnostic cutoff. This isolated deviation was caused by inherent threshold-related detection fluctuation rather than systematic false-positive bias of the assay. Overall, the favorable specificity of pVNA largely mitigates clinical misdiagnosis risk, ensuring credible serological assessment and preventing redundant post-exposure intervention. Notably, although pVNA performs well using the WHO-recommended 0.5 IU/mL threshold, direct cross-method extrapolation of cutoff values remains controversial. Moore SM (48) argued that the cutoff values should be independently determined when investigating the applicability of the recognized 0.5 IU/mL cutoff value between different methods, which is not extrapolated. Further research is needed to address this issue in the future.

Theoretically, optimal sensitivity and specificity reach 100%. Due to the defects of the screening method itself and the complexity of biological samples, many physiological parameters, including the parameter ranges of negative and positive individuals, cross and overlap (49). Compared with the commercial ELISA, the constructed pVNA consistently analyzed the attenuation trend of antibody levels in the population. The positive coincidence rate in pVNA and ELISA was 95.28%, and the linear analysis yielded consistent results. Debnath et al. (50, 51) reported that the sensitivity of ELISA was 94.4%, and the specificity was 95.2%, when the sample value was within the limit range. These findings were similar to the indicators of the RFFIT assay, and the results were highly consistent and correlated. Although the use of commercially available ELISA kits is quick and easy, the kits can only detect serum values in the effective linear range from 0.5 IU/mL to 10 IU/mL (52), and high false-positive and false-negative rates are noted for some out-of-range serum samples. Therefore, this assay can be used to evaluate the trend of rabies antibodies. Unlike commercial ELISA with a narrow linear range, pVNA covers a wider titer spectrum for accurate neutralizing antibody quantification. The log ED_50_ quantitative range of 2.80–4.26 was calibrated with WHO reference immunoglobulin to define the pVNA’s quantification interval. Samples within this range achieve precise antibody quantification with qualified intra-assay precision. This dilution-based 4PL range corresponds to the linear regression range of 0.09–93.73 IU/mL, where log-transformed concentration and log ED_50_ values maintain strict linear proportionality. The two parameters describe distinct metrics. The log ED_50_ 2.80–4.26 range reflects acceptable dilution folds when ED_50_ are calculated by nonlinear sigmoidal 4PL, and is used to determine the upper and lower detection limits. Whereas 0.09–93.73 IU/mL is the concentration range satisfying linearity criteria via linear regression. Notably, the linear lower bound of 0.09 IU/mL does not equal the clinical limit of detection of 2.63 IU/mL. The 0.09 IU/mL cutoff is derived from purified reference material for standard linearity quantification, while the LOD of 2.63 IU/mL, determined using positive human sera, marks the lowest antibody concentration distinguishable from negative background to confirm sample seropositivity. In addition, both FAVN (53) and RFFIT (54) require the use of RABV CVS-11, which involves biosafety (55), thus limiting the application of these assays in rabies postimmunization evaluation. Compared with the detection times of RFFIT/FAVN (72 h) and MNT (14 d), pVNA can be performed within 48 h, demonstrating significant advantages over traditional methods.

This study focused on PEP populations, but the pVNA, ELISA, and RFFIT assays used herein are technically applicable to pre-exposure (PrEP) populations. These assays target rabies G-protein-specific neutralizing antibodies, which are induced by identical vaccine antigens in PrEP and PEP schedules. While antibody dynamics (peak titer, decay rate) may differ among the three assays due to distinct vaccination regimens, their ability to detect and quantify protective antibodies remains consistent, as supported by WHO guidelines (56) and prior studies (57). Future studies could extend our findings by evaluating PrEP populations to confirm these trends.

In conclusion, based on the above-mentioned RABV neutralizing antibody detection assays, the pVNA developed in this study showed broad robustness, was less time-consuming and had reduced biosafety demands. It can be used to evaluate the serological characteristics of healthy individuals either exposed to RABV or vaccinated with vaccines. This feature will aid in modeling the dynamic immune response after RABV vaccination and provide evidence for enhancing vaccination strategies.

## SUPPLEMENTAL MATERIAL

Supplemental figure legends jcm.00877-26-s0001.docx Legends for Fig. S1 to S3.

Figure S1 jcm.00877-26-s0002.tiff RABVG protein expression in infected cells was verified by Western blot with anti-RABV G primary antibody.

Figure S2 jcm.00877-26-s0003.tiff The number of fluorescent spots in the 800T pXN2‑RABV‑G pseudovirus varied with time, and was the highest at 48 h.

Figure S3 jcm.00877-26-s0004.tiff Diagnostic plots for the generalized additive models (GAM) fitted to neutralizing antibody decay data from 4-dose and 5-dose cohorts, measured by RFFIT and pVNA. Panels include residual vs predicted values, homoscedasticity plots, Q-Q plots for residual normality, and residual vs order plots, confirming valid model assumptions across all groups.

Supplemental tables jcm.00877-26-s0005.docx Tables S1 to S11.

ASM does not own the copyrights to Supplemental Material that may be linked to, or accessed through, an article. The authors have granted ASM a non-exclusive, world-wide license to publish the Supplemental Material files. Please contact the corresponding author directly for reuse.
