# Supplementary material for: A pseudovirus-based neutralizing antibody assay for measuring neutralizing antibody against rabies virus
Source: J Clin Microbiol. 2026 Aug 21;64(9):e00877-26. doi: 10.1128/jcm.00877-26 (PMC13556208; doi:10.1128/jcm.00877-26)
Supplement: Supplementary file 1 [file jcm.00877-26-s0001.docx]

Supplement Figure 1: RABV G protein expression in infected cells was verified by Western blot with anti-RABV G primary antibody.

Supplement Figure 2: The number of fluorescent spots in the 800T pXN2-RABV-G pseudovirus varied with time, and was the highest at 48h.

Supplement Figure 3: Diagnostic plots for the generalized additive models (GAM) fitted to neutralizing antibody decay data from 4-dose and 5-dose cohorts, measured by RFFIT and pVNA. Panels include residual vs. predicted values, homoscedasticity plots, Q-Q plots for residual normality, and residual vs. order plots, confirming valid model assumptions across all groups.
