# Supplementary material for: A pseudovirus-based neutralizing antibody assay for measuring neutralizing antibody against rabies virus
Source: J Clin Microbiol. 2026 Aug 21;64(9):e00877-26. doi: 10.1128/jcm.00877-26 (PMC13556208; doi:10.1128/jcm.00877-26)
Supplement: Supplementary file 2 [file jcm.00877-26-s0005.docx]

Supplemental table 1: Cost-effectiveness analysis of laboratory testing methods after rabies vaccination.

| **Methods for laboratory testing after rabies vaccination** | **Advantage** | **Disadvantage** | **Testing time** | **Cost** |
| --- | --- | --- | --- | --- |
| Mouse neutralization test (MNT) | Serial passage of virus from mouse brain reduced viral virulence. | The repeatability, consistency and sensitivity were poor. | 14 days | Approximate $8500 / 100 samples |
| Rapid fluorescent focus inhibition test (RFFIT) | The gold standard recommended by WHO for antibody titer detection has high accuracy. | It requires the use of highly virulent strains, which can only be operated in P3 laboratories, and requires expensive fluorescence detection equipment. | 2 days | Approximate $1,500 / 100 samples |
| Fluorescent antibody virus neutralization test (FAVN) | The standard method recommended by OIE for the detection of neutralizing antibodies against rabies virus in animals is highly accurate. | Positive standards are needed, and the import is difficult and demanding. | 7 days | Approximate $14,000 / 100 samples |
| Enzyme linked immunosorbent assay (ELISA) | The detection of total IgG antibody is fast, automatic, does not use real virus and has high safety. | It can only detect serum values in the effective linear range of 0.5IU/ml-10 IU/ml, with high false positive and false negative rates. | 1 days | Approximate $4,200 / 100 samples |
| Pseudovirus neutralising antibody test (PVNA) | It does not use real virus, high safety, easy to obtain pseudovirus, simple operation, low cost, short experimental time, high sensitivity, high accuracy, and can detect specific antibodies. | Only neutralizing antibody titers against specific proteins can be assessed. | 2 days | Approximate $700.00 / 100 samples |

Supplemental table 2: Polymerase Chain Reaction (PCR) Primer Sequences.

VSV-RBAV-GFP F (MluI)

5'-ACAGAGATCGATCTGTTTACGCGTCACTATGGTTCCTCAGGCTCTCCTGT-'3

The Mlu I restriction site of the F primer inserted for RBAV

VSV- RBAV-GFP R (Xho I)

5'-CGCCCTTGCTCACCATCTCGAGCGTGATATCTGTTAGTTTTTTTCATACCTAGCAGGATTTGAGTCACAGTCCGGTCTCACCCC-'3

The Xho I restriction site of the R primer inserted for RBAV

Supplemental table 3: Results of lesions after infected cells with virus dilution for 48h.

| Virus dilution | CPE^a^ | NO CPE | Total | | CPE Percentage (%) |
| --- | --- | --- | --- | --- | --- |
|  |  |  | CPE | NO CPE |  |
| 10^-1^ | 8 | 0 | 27 | 0 | 100(27/27) |
| 10^-2^ | 8 | 0 | 19 | 0 | 100(19/19) |
| 10^-3^ | 7 | 1 | 11 | 1 | 91.6(11/12) |
| 10^-4^ | 3 | 5 | 4 | 6 | 40(4/10) |
| 10^-5^ | 1 | 7 | 1 | 13 | 0.7(1/14) |
| 10^-6^ | 0 | 8 | 0 | 21 | 0(0/21) |
| 10^-7^ |  |  |  |  |  |

a: cytopathic effect (CPE), the number of Wells positive for virus dilution.

Supplemental table 4: Results of repeatability tests on 10 samples.

| Sample | Sample classification | $\overline{x}$ (IU/mL) | s | cv% |  |
| --- | --- | --- | --- | --- | --- |
| R1 | Enhanced positivity | 22.640 | 2.791 | 12.33% |  |
| R2 | Enhanced positivity | 11.865 | 1.492 | 12.58% |  |
| R3 | Enhanced positivity | 8.910 | 0.958 | 10.75% |  |
| R4 | Enhanced positivity | 9.122 | 0.597 | 6.54% |  |
| R5 | Moderately positive | 1.624 | 0.218 | 13.44% |  |
| R6 | Moderately positive | 4.387 | 0.514 | 11.72% |  |
| R7 | Moderately positive | 0.921 | 0.120 | 13.06% |  |
| R8 | Moderately positive | 2.750 | 0.301 | 10.95% |  |
| R9 | Weakly positive | 0.669 | 0.101 | 15.13% |  |
| R10 | Weakly positive | 0.549 | 0.042 | 16.83% |  |

Supplemental table 5: Results of the samples examined by operator A/B for six working days.

| Sample | Experimenter A | | | | |  | Experimenter B | | | | |
| --- | --- | --- | --- | --- | --- | --- | --- | --- | --- | --- | --- |
|  | $\overline{x}$ (IU/mL) | s | CV%^a^ | χ2 | P |  | $\overline{x}$ (IU/mL) | s | CV% | χ2 | P |
| R1 | 24.11 | 0.70 | 2.89% | 9.41 | 0.09 |  | 24.17 | 0.83 | 3.42% | 1.91 | 0.86 |
| R2 | 12.05 | 1.66 | 13.80% |  |  |  | 12.07 | 1.53 | 12.71% |  |  |
| R3 | 8.89 | 0.41 | 4.56% |  |  |  | 9.21 | 0.68 | 7.35% |  |  |
| R4 | 9.42 | 0.87 | 9.26% |  |  |  | 9.53 | 0.89 | 9.36% |  |  |
| R5 | 1.62 | 0.14 | 8.55% |  |  |  | 1.55 | 0.18 | 11.57% |  |  |
| R6 | 4.08 | 0.74 | 18.20% |  |  |  | 4.13 | 0.75 | 18.24% |  |  |
| R7 | 0.82 | 0.09 | 11.59% |  |  |  | 0.81 | 0.05 | 6.68% |  |  |
| R8 | 2.98 | 0.20 | 6.64% |  |  |  | 2.92 | 0.23 | 7.88% |  |  |
| R9 | 0.87 | 0.27 | 31.23% |  |  |  | 0.94 | 0.27 | 28.30% |  |  |
| R10 | 0.56 | 0.04 | 13.74% |  |  |  | 0.55 | 0.02 | 10.09% |  |  |

CV% is the coefficient of variation of the same sample tested by operator A/B over 6 working days, and χ2 is the difference between the populations tested by operator A/B over 6 days.

Supplemental table 6: Tesults of 10 samples were tested by experimenters A and B on six working days.

| Sample | Day-1 | | | Day-2 | | | Day-3 | | | Day-4 | | | Day-5 | | | Day-6 | | |
| --- | --- | --- | --- | --- | --- | --- | --- | --- | --- | --- | --- | --- | --- | --- | --- | --- | --- | --- |
|  | CV%^a^ | Z | P | CV% | Z | P | CV% | Z | P | CV% | Z | P | CV% | Z | P | CV% | Z | P |
| R1 | 3.90% | 22 | 0.58 | 0.73% | 27 | 0.60 | 5.21% | 23 | 0.65 | 1.37% | 14 | 0.17 | 0.47% | 16 | 0.24 | 0.22% | 24 | 0.72 |
| R2 | 1.23% |  |  | 2.77% |  |  | 6.84% |  |  | 0.28% |  |  | 6.26% |  |  | 2.08% |  |  |
| R3 | 3.25% |  |  | 5.38% |  |  | 6.60% |  |  | 12.86% |  |  | 0.33% |  |  | 2.72% |  |  |
| R4 | 0.93% |  |  | 0.89% |  |  | 1.71% |  |  | 5.41% |  |  | 1.03% |  |  | 0.24% |  |  |
| R5 | 5.27% |  |  | 7.62% |  |  | 5.34% |  |  | 10.55% |  |  | 2.32% |  |  | 2.24% |  |  |
| R6 | 6.16% |  |  | 2.61% |  |  | 5.43% |  |  | 6.02% |  |  | 6.19% |  |  | 1.72% |  |  |
| R7 | 9.32% |  |  | 1.02% |  |  | 7.57% |  |  | 7.53% |  |  | 7.71% |  |  | 3.11% |  |  |
| R8 | 4.69% |  |  | 2.04% |  |  | 1.16% |  |  | 5.07% |  |  | 3.71% |  |  | 6.85% |  |  |
| R9 | 2.92% |  |  | 4.43% |  |  | 8.49% |  |  | 11.29% |  |  | 7.05% |  |  | 15.20% |  |  |
| R10 | 17.51% |  |  | 11.67% |  |  | 1.75% |  |  | 14.68% |  |  | 4.58% |  |  | 6.88% |  |  |

a: CV% is the coefficient of variation for operators A and B to detect the same sample on the same day, and Z is the difference between operators A and B to detect the population on the same day.

Supplemental table 7: Effect of culture medium and cell passage on sample titer .

|  | Medium within 1 months  after production | Medium within 6 months  after production | Medium within 12 months  after production | CV% | χ2 | P |
| --- | --- | --- | --- | --- | --- | --- |
| N1 | 5.20 ± 0.10 | 4.91 ± 0.14 | 4.90 ± 0.12 | 3.60% | 4.00 | 0.14 |
| N2 | 3.78 ± 0.24 | 3.68 ± 0.12 | 3.70 ± 0.06 | 3.88% |  |  |
| N3 | 1.91 ± 0.08 | 2.02 ± 0.08 | 1.95 ± 0.10 | 4.65% |  |  |
| N4 | 10.49 ± 0.10 | 11.39 ± 0.36 | 10.35 ± 0.83 | 6.23% |  |  |
| N5 | 2.33 ± 0.01 | 2.16 ± 0.11 | 2.32 ± 0.21 | 6.44% |  |  |
| N6 | 3.46 ± 0.08 | 3.12 ± 0.52 | 2.72 ± 0.20 | 13.79% |  |  |
| N7 | 1.12 ± 0.01 | 1.01 ± 0.03 | 1.12 ± 0.03 | 5.46% |  |  |
| N8 | 0.22 ± 0.00 | 0.24 ± 0.01 | 0.10 ± 0.01 | 33.98% |  |  |
| GMT (IU/mL) | 2.30 | 2.26 | 2.01 | - |  |  |
|  | Passages 3 | Passages 5 | Passages 7 | CV% | χ2 | P |
| N1 | 5.20 ± 0.10 | 4.67 ± 0.23 | 4.70 ± 0.40 | 7.20% | 0.25 | 0.88 |
| N2 | 3.78 ± 0.24 | 3.70 ± 0.08 | 3.17 ± 0.30 | 9.82% |  |  |
| N3 | 1.91 ± 0.08 | 2.03 ± 0.08 | 2.04 ± 0.19 | 6.41% |  |  |
| N4 | 10.49 ± 0.10 | 11.22 ± 0.47 | 10.53 ± 0.30 | 4.24% |  |  |
| N5 | 2.33 ± 0.01 | 2.15 ± 0.11 | 1.85 ± 0.14 | 10.93% |  |  |
| N6 | 3.46 ± 0.08 | 3.12 ± 0.51 | 2.72 ± 0.07 | 13.38% |  |  |
| N7 | 1.12 ± 0.01 | 1.05 ± 0.04 | 1.44 ± 0.32 | 19.90% |  |  |
| N8 | 0.22 ± 0.00 | 0.24 ± 0.01 | 0.24 ± 0.03 | 7.42% |  |  |
| GMT  (IU/mL) | 2.30 | 2.25 | 2.20 | - |  |  |

Supplemental table 8: Four-parameter logistic fitting statistics of four replicates for International Standard for Anti-Rabies Immunoglobulin from 0.64 to 164 IU/mL.

| Parameter | Repeat 1 | Repeat 2 | Repeat 3 | Repeat 4 | Mean |
| --- | --- | --- | --- | --- | --- |
| Top (maximum respone signal) | 23327 | 21275 | 24848 | 20101 | 22387.75 |
| Bottom (baseline signal) | 627.8 | 640.2 | 659.4 | 673.8 | 650.30 |
| log _(Bottom)_ | 2.80 | 2.81 | 2.82 | 2.83 | 2.81 |
| Span = Top - Bottom | 22699 | 20635 | 24188 | 19427 | 21737.25 |
| log _(Span)_ | 4.36 | 4.31 | 4.38 | 4.29 | 4.34 |
| Yupper = Bottom + 0.9 × Span | 21057.08 | 19211.52 | 22429.14 | 18158.28 | - |
| log _(Yupper)_ | 4.32 | 4.28 | 4.35 | 4.26 | - |
| logIC_50_ | 1.709 | 1.675 | 1.713 | 1.65 | 1.69 |
| HillSlope | 2.105 | 2.129 | 2.105 | 2.316 | 2.16 |
| IC_50_ | 51.21 | 47.34 | 51.66 | 44.69 | 48.73 |
| Degrees of freedom | 5 | 5 | 5 | 5 | - |
| R^2^ | 0.9641 | 0.9552 | 0.9729 | 0.953 | - |
| Sum of Squares | 19263705 | 21216873 | 16305079 | 21260866 | - |
| Sy.x | 1963 | 2060 | 1806 | 2062 | - |

Supplemental table 9: Effect of sample cycles on sample titer.

|  | GMT (IU/mL) | χ2 | P |  | Between-group variance | Z | P |
| --- | --- | --- | --- | --- | --- | --- | --- |
| Non-freeze samples | 13.30 | 30.54 | <0.00 |  | Non-freeze samples vs.5 cycles samples | 5.379 | <0.00 |
| 1 cycle samples | 11.80 |  |  |  | Non-freeze samples vs.3 cycles samples | 2.749 | 0.04 |
| 3 cycles samples | 10.53 |  |  |  | Non-freeze samples vs.1 cycle samples | 3.705 | <0.01 |
| 5 cycles samples | 8.91 |  |  |  |  |  |  |

Supplemental table 10: Effect of region and sampling time on sample titer.

|  | Sample | GMT (IU/mL) | CV% | *U/H* | P |
| --- | --- | --- | --- | --- | --- |
| Geographical | R1 | 82.65 | 0.43% | 12.00 | 0.92 |
| HeYuan | R2 | 46.53 | 12.73% |  |  |
|  | R3 | 6.01 | 1.61% |  |  |
|  | R4 | 83.66 | 7.53% |  |  |
|  | R5 | 15.95 | 4.21% |  |  |
| PanYu | R6 | 31.10 | 0.27% |  |  |
|  | R7 | 70.73 | 2.70% |  |  |
|  | R8 | 53.91 | 2.02% |  |  |
|  | R9 | 32.69 | 1.40% |  |  |
|  | R10 | 18.17 | 0.29% |  |  |
| Samples collected |  |  |  |  |  |
| 2020 | T1 | 38.57 | 0.64% | 0.96 | 0.81 |
|  | T2 | 3.50 | 5.05% |  |  |
| 2021 | T3 | 61.75 | 0.00% |  |  |
|  | T4 | 14.30 | 3.87% |  |  |
|  | T5 | 27.20 | 0.55% |  |  |
| 2022 | T6 | 39.82 | 0.83% |  |  |
|  | T7 | 48.32 | 2.85% |  |  |
|  | T8 | 11.26 | 0.82% |  |  |
| 2023 | T9 | 61.75 | 0.00% |  |  |
|  | T10 | 14.89 | 0.71% |  |  |
|  | T11 | 36.42 | 0.59% |  |  |

Supplemental table 11: Detection limit of 5 high titer samples.

| Dilution ratio | D1 | D2 | D3 | D4 |
| --- | --- | --- | --- | --- |
|  | Detection rates (%) | Detection rates (%) | Detection rates (%) | Detection rates (%) |
| 1:64 | (12/12)100.00% | (12/12)100.00% | (12/12)100.00% | (12/12)100.00% |
| 1:128 | (12/12)100.00% | (12/12)100.00% | (12/12)100.00% | (12/12)100.00% |
| 1:256 | (11/12)91.67% | (11/12)91.67% | (10/12)83.33% | (8/12)66.67% |
| 1:512 | (7/12)58.33% | (9/12)75.00% | (7/12)58.33% | (3/12)25.00% |
| 1:1024 | (2/12)16.67% | (5/12)41.67% | (2/12)16.67% | (0/12)0.00% |
| 1:2048 | (0/12)0.00% | (0/12)0.00% | (0/12)0.00% | (0/12)0.00% |
| 1:4096 | (0/12)0.00% | (0/12)0.00% | (0/12)0.00% | (0/12)0.00% |
| 1:8192 | (0/12)0.00% | (0/12)0.00% | (0/12)0.00% | (0/12)0.00% |
